# A university career in basic and applied avian immunology: important contributions of chicken models for autoimmune diseases

**DOI:** 10.3389/fphys.2026.1797637

**Published:** 2026-02-11

**Authors:** Gisela F. Erf

**Affiliations:** Department of Poultry Science, University of Arkansas System Division of Agriculture, Fayetteville, AR, United States

**Keywords:** avian immunology, cell-mediated immunity, growing feather bioassay, inflammatory response, obese strain Hashimoto’s thyroiditis, Smyth line autoimmune vitiligo, UCD200/206 scleroderma/systemic sclerosis

## Introduction

It is an honor to be invited to contribute an opinion paper for the Frontiers in Avian Physiology Lifetime Achievements Topic and have the opportunity to share and reflect on my 38 year career in academia as a researcher, teacher, and mentor, and to bring awareness to the importance of genetic lines of chickens to unravel complex interactions of the immune system with other physiological systems.

## Educational and professional journey

I grew up in southwestern Germany. Having no clear plans after High School (Abitur), I spent the summer with family friends in New Brunswick, Canada. That summer turned into now 50 years in North America.

After 1 year studying science at the University of New Brunswick, I realized I needed to find real-life applications to my studies. Through friends, I learned about the Nova Scotia Agricultural College and the many science-based problem solving contributions one could make in agricultural sciences. After graduating with an Associate Degree in Animal Science from NSAC, I enrolled at the University of Guelph to complete the BS degree. One of my favorite classes was reproductive physiology taught by Dr. Robert Etches. During office hours, he encouraged me to pursue an MS degree under his tutelage working on understanding the ovulation cycle of the hen. I happily accepted and much enjoyed working with chickens and in the laboratory, using radioimmunoassays to establish hormone profiles leading to ovulation, learning endocrinology and reproductive physiology, as well as studying the literature, writing, and presenting and publishing our novel research findings (Lang et al., 1984a; Lang et al., 1984b). Moreover, I greatly value this opportunity and introduction to basic scientific research and its real-life application in an important production animal.

As I was nearing completion of the MS degree, I knew I wanted to pursue a PhD. An opportunity presented itself during a brief visit to Cornell University where I met Dr. James A. Marsh, Avian Immunologist, who was looking for a graduate student interested in working on neuro-endocrine-immune interactions in chickens. I had no background in immunology, but the opportunity to combine my MS experience with a new scientific field piqued my interest. I applied and was accepted into Cornell’s newly formed Inter-college Immunology PhD program under the tutelage of Dr. Marsh in the Department of Poultry Science, supported by a teaching assistantship in Biology. The learning and training environments in the Department of Poultry Science, the Immunology program, and the Biology Department were superb. The immunology program offered an introductory course that provided me a solid basis for the excellent, intensive graduate courses on various immunology topics. The most impactful course for me was immunophysiology which was taught by my mentor, Dr. Marsh. He introduced us to the many interactions between the immune system and other physiological systems, and to the eloquent experimental approaches used to demonstrate these interactions in avian models. He deeply instilled in me to view the immune system in the “whole animal” context and not as a system working independently of other physiological systems. This philosophy played a major role in my future research and teaching, as well as in my ability to collaborate with nutritionists, geneticists, physiologists, environmental scientists, veterinarians, and biomedical researchers on immunology projects.

For my dissertation, I studied thyroid-immune system interactions using chicken strains with hormonal abnormalities. Most experiments focused on the sex-linked recessive dwarf (SLD) strain in comparison to the normal growing Cornell K (K) strain control. The SLD-strain has near-normal thyroid activity but impaired peripheral conversion of thyroxine (T4) to triiodothyronine (T3) due to low peripheral 5′-monodeiodinase activity. The K and SLD strains were originally derived from the same populations and, importantly for comparative immune function studies, both strains were homozygous for *B*15 at the major histocompatibility complex--the main genetic region coding for immunoregulatory genes. Using the chorio-allantoic membrane (CAM) bioassay and the mixed lymphocyte response (MLR) culture assay to measure alloantigen-reactive lymphocyte responses, we found that this response was lower in the SLD-than the K-strain throughout a 20-month study. Dietary supplementation of T3 increased plasma concentrations of T3 in both strains but did not enhance the SLD strain’s depressed alloantigen responses. On the other hand, using *in vitro* mitogen proliferation assays, the responses to the T cell mitogens phytohemagglutinin (PHA) and concanavalin A (Con A) were not different in SLD-compared to K-strain cockerels and increased with increasing dietary T3 supplementation in both strains. However, the PHA:ConA ratio (PHA and Con A activate different T cell subsets) was lower in SLD-than in K-strain chickens at 6 and 12 week but increased to K-strain levels by 12 weeks with T3 supplementation. Collectively, these findings support immune-modulating effects of T3 on cell-mediated immune responses, whereby effects depended on the age of the chicken, the T cell population stimulated, and method of T cell activation, i.e., through the T cell receptor in the CAM and MLR assays versus mitogen receptors. I should mention that I developed all the assays for these studies, which was a challenge and often led to frustration, even to the point of considering a project change. But in the end, everything came together in a comprehensive dissertation and four peer-reviewed publications, as well as a book chapter co-authored with Dr. Marsh (Erf et al., 1987; Erf and Marsh, 1987; Erf and Marsh, 1988; Erf and Marsh, 1989; Marsh and Erf, 1996).

Upon graduation, I accepted a tenure-track position as Assistant Professor (Immunology) in Biological Sciences at Smith College, a Liberal Arts College for women in Northampton, MA. Having discovered my passion for teaching at Cornell University and gained a well-rounded knowledge-base in immunology, I felt well prepared for this position. Despite the heavy teaching appointment, I was able to set up a research project on immune system development in congenitally hypothyroid mice (Erf, 1993), for which I received an NSF Research Opportunities for Women, Research Planning Grant. An invitation to collaborate from Dr. J. Robert Smyth, Jr, Poultry Geneticist at the University of Massachusetts, Amherst, provided a return to avian immunology. Dr. Smyth had developed a chicken model characterized by spontaneous, post-natal loss of pigment cells (melanocytes) in feathers and eyes, and suspected that the immune system played a role in the melanocyte loss. With his mentorship, I prepared and was awarded an NIH R15 grant to study the role of T cells in the loss of epidermal melanocytes in what is now known as the Smyth line chicken model for autoimmune vitiligo. With my team of outstanding undergraduate researcher we made great progress, defining T cell population profiles in growing feathers (site of epidermal pigment cells) before and throughout out progression of vitiligo in the same individual, which was possible due to the predictable disease expression and the minimally invasive access to the target tissue (Erf et al., 1995; Shresta et al., 1997; Erf et al., 1997). This project reignited my passion for research, the chicken model, working with students in the laboratory and at the farm, attending professional meetings, and immersing myself in the research topic. However, I was spending more and more time at work, and realized I needed a better research-teaching and life-work balance, especially after the birth of our son.

At this time, I was made aware that the newly formed Department and Center of Excellence for Poultry Science at the University of Arkansas in Fayetteville, AR, was looking for an avian immunologist. I applied, hoping that 5 years of teaching experience and building a successful research program in immunology at an undergraduate institution would still make me competitive for this position. I got the job! and for the first few years felt like I was on sabbatical! It was a dream come true to work within a state-of-the-art research facility, in a large department focused on poultry science at all levels, with outstanding, supportive colleagues to learn from and collaborate with, and with opportunities to mentor both undergraduate and graduate student research. To meet my 20% teaching appointment, I developed a graduate level immunology lecture course, an intensive immunology laboratory course, and an undergraduate hands-on research course, which was funded by a USDA Higher Education Challenge grant. For my research I established breeding populations of the Smyth line vitiligo model, which consists of three MHC-matched lines, i.e.: 1) the vitiligo-susceptible, highly disease expressing Smyth line (65%–95% vitiligo incidence; varied low incidence of associated autoimmune diseases like uveitis, alopecia areata and hypothyroidism), 2) the vitiligo-susceptible but low expressing parental Massachusetts Brown line (0%–2% incidence), and 3) the Light-brown Leghorn, which serves as the non-susceptible, pigmentation control (Sreekumar et al., 1996; Wick et al., 2006; Erf, 2021). We received several federal grants for research on autoimmune vitiligo and for other multifactorial diseases using chicken models, e.g., the Pulmonary Arterial Hypertension (ascites) Resistant and Susceptible lines (Pavlidis et al., 2007; Hamal et al., 2010a; Hamal et al., 2010b; Wideman et al., 2013). My efforts in immunology research and teaching were recognized through UA and national awards, as well as an Endowed Professorship in Avian Immunology from Tyson Foods, which enabled me to provide numerous experiential learning opportunities for undergraduates working with our animal models. These efforts greatly fostered the students’ enthusiasm for learning and pursuing future careers in academia, veterinary and human medicine, and the Poultry and Allied Industry.

In 2015, we adopted the last remaining populations of UCD 200/206 chickens with autoimmune scleroderma/systemic sclerosis (UCD-SSc; a fibrotic disease of skin and organs with underlying vasculitis), as well as the Obese strain (OS) of chickens with Hashimoto’s thyroiditis. Like the Smyth line autoimmune vitiligo model, the UCD-SSc and OS chickens made, and continue to make, important contributions to avian immunology and our understanding of the multifactorial nature of autoimmune and other postnatal diseases (Wick et al., 2006; Erf and Le Poole, 2019; Erf, 2021). For all avian autoimmune models, disease susceptibility was shown to be multigenic, resulting in aberrant target cell-, immune-, and/or endocrine-functions that may not result in disease without extrinsic and/or intrinsic environmental factors (Wick et al., 2006; Erf, 2021).

We also received grants from the Poultry and Allied Industry, including funding for studies on maternal antibodies (Hamal et al., 2006), the effects of nutrition, genetic selections, heat- and cold-stress, dietary immunomodulators on the immune system, as well as immune responses to vaccines and immunomodulatory effects of vaccine adjuvants (Bottje et al., 1997; Fritts et al., 2004; Konjufca et al., 2004; Wideman et al., 2004; Bowen et al., 2006; Rocchi et al., 2023; Santamaria et al., 2024; Beck et al., 2025). Through our research efforts, teaching immunology, mentoring students at all levels, and collaborations with colleagues in different fields, I never stopped learning--keeping up with the extensive and fast moving field of immunology, experimental techniques and approaches, and studying various animal, organ, and cellular systems.

## Contributions of Smyth line autoimmune vitiligo studies to avian immunology

Throughout the years, we established the Smyth line autoimmune vitiligo model as an excellent model for human autoimmune vitiligo and associated autoimmune diseases for biomedical and translational research. Additionally, by studying immune system development and function in healthy controls and disease-susceptible individuals, we contributed a lot of new knowledge and experimental approaches to avian immunology, especially regarding cellular immunity (Wang and Erf, 2003; Wang and Erf, 2004; Shi et al., 2012; Shi and Erf, 2012; Jang et al., 2014; Sorrick et al., 2022; Falcon et al., 2024).

One of the most impactful methods that originated when studying the evolving autoimmune response to epidermal melanocytes in the pulp of growing feathers (GFs), is the GF-pulp bioassay. The GF-pulp is a column (e.g., 8–10 by 2–3 mm) of cutaneous tissue which consists of mostly inner dermis and an outer epidermal layer and is surrounded by the feather sheath. The GFs are loosely attached in the follicular cup, can be easily removed, regenerate, and yield sufficient pulp tissue for multiple *ex vivo* analyses. Simultaneous intradermal (i.d.) injection of a test-material (e.g., microbial components, antigens, vaccines, etc.) into multiple GFs of an individual, and subsequent periodic collection of GFs for laboratory analysis, allows for quantitative, qualitative, and temporal assessment of the *in vivo* cellular/tissue responses to the test-material. This is comparable to the periodic sampling of blood to measure humoral responses, except each GF constitutes a defined reaction site–hence, we often refer to it as an “*in vivo* test tube.” This approach revealed differences in inflammatory responses to different microbial components, vaccine antigens, mitogens, bioactivities of nanomaterials, and adjuvants, and was shown to provide a unique platform to uncover effects of nutrition, environment, and genetic selection on cellular responses initiated in GF-pulps in chickens, and, more recently, in turkeys. Moreover, the combination of the GF bioassay with other minimally invasive procedures (e.g., blood sampling, mucosal swabs) to collect samples for *ex vivo* and *in vitro* laboratory analyses, yields insight into local and systemic immune responses to the i.d. injected test-material (Erf and Ramachandran, 2016; Sullivan and Erf, 2017; Erf et al., 2017; Erf et al., 2023; Rocchi et al., 2023; Byrne and Erf, 2024; Beck et al., 2025; Santamaria et al., 2025; Anderson et al., 2025). This approach greatly reduces the number of animals needed to study tissue/cellular responses over time from their initiation to resolution in the “whole animal” context.

Looking back, I am very excited and thankful to have had such a full-filling career in research and teaching and the opportunity to contribute to the advancement of avian immunology. I was fortunate to have access to the avian autoimmune disease models and highly value their contributions to my career, the professional development of many undergraduate and graduate students, and our understanding of avian immune system development and function in health and disease.
